# Intrinsic mechanism and pharmacologic treatments of noise-induced hearing loss

**DOI:** 10.7150/thno.83383

**Published:** 2023-06-19

**Authors:** Ke Xu, Baoying Xu, Jiayi Gu, Xueling Wang, Dehong Yu, Yu Chen

**Affiliations:** 1 Materdicine Lab, School of Life Sciences, Shanghai University, Shanghai, China.; 2 Department of Otolaryngology-Head and Neck Surgery, Shanghai Ninth People's Hospital, School of Medicine, Shanghai Jiao Tong University, Shanghai, China.; 3 Ear Institute, School of Medicine, Shanghai Jiao Tong University, Shanghai, China.; 4 Shanghai Key Laboratory of Translational Medicine on Ear and Nose Diseases, Shanghai, China.

**Keywords:** noise-induced hearing loss, pharmacologic strategies, drug delivery systems, nanoparticles

## Abstract

Noise accounts for one-third of hearing loss worldwide. Regretfully, noise-induced hearing loss (NIHL) is deemed to be irreversible due to the elusive pathogenic mechanisms that have not been fully elucidated. The complex interaction between genetic and environmental factors, which influences numerous downstream molecular and cellular events, contributes to the NIHL. In clinical settings, there are no effective therapeutic drugs other than steroids, which are the only treatment option for patients with NIHL. Therefore, the need for treatment of NIHL that is currently unmet, along with recent progress in our understanding of the underlying regulatory mechanisms, has led to a lot of new literatures focusing on this therapeutic field. The emergence of novel technologies that modify local drug delivery to the inner ear has led to the development of promising therapeutic approaches, which are currently under clinical investigation. In this comprehensive review, we focus on outlining and analyzing the basics and potential therapeutics of NIHL, as well as the application of biomaterials and nanomedicines in inner ear drug delivery. The objective of this review is to provide an incentive for NIHL's fundamental research and future clinical translation.

## Introduction

**Figure 1:**
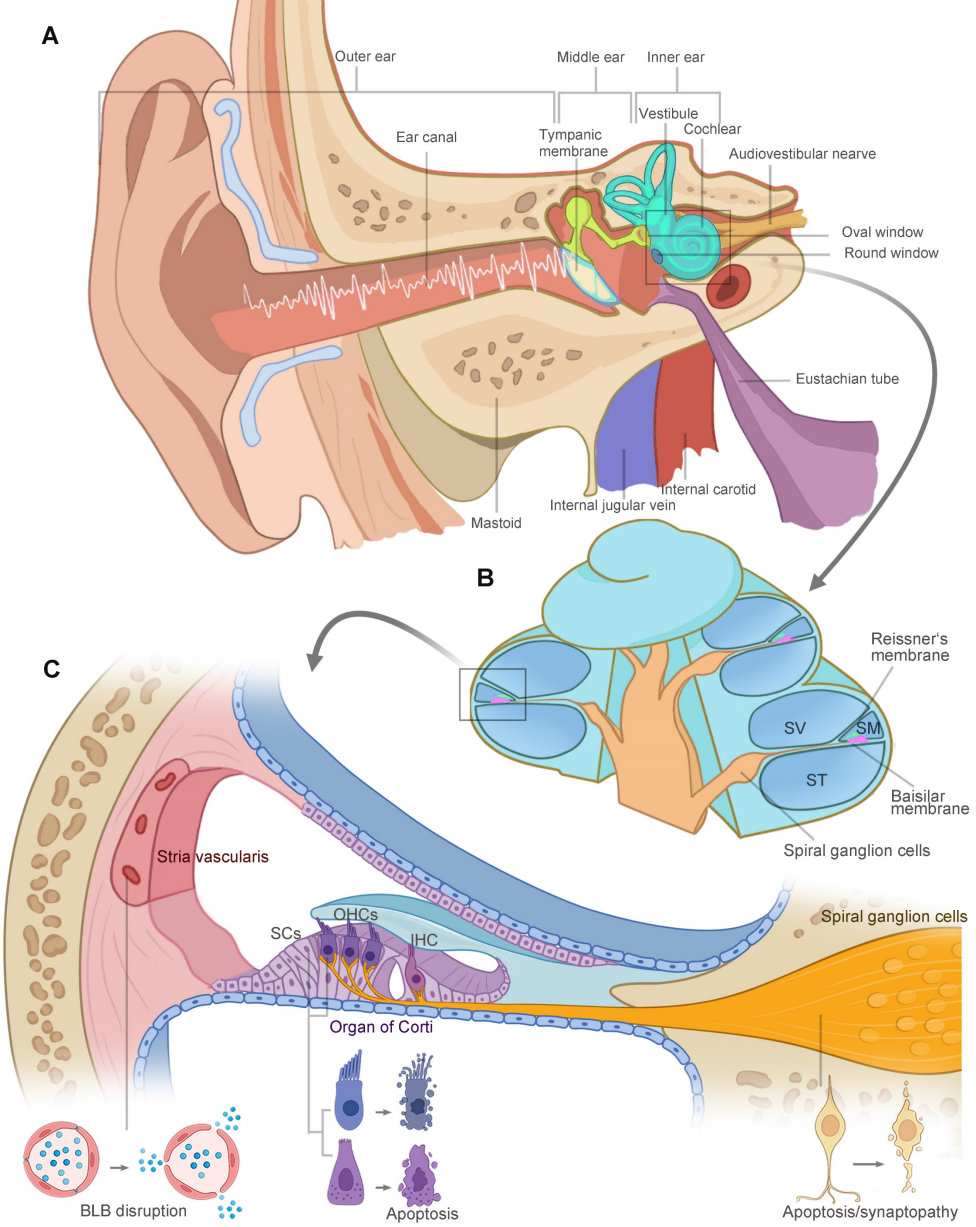
** The anatomy of the human ear. (A)** Schematic representation of ear anatomy. The ear consists of three parts: the outer, middle, and inner ear. The outer and middle ear are separated by the tympanic membrane. Sound waves are transduced through tympanic membrane to the chain of three tiny bones (the ossicles) in middle ear cavity, which is attached to the oval window membrane, leading to fluid vibrations in the inner ear. The inner ear is responsible for hearing (cochlea) and balance (vestibule). **(B)** The cochlea is divided into three fluid-filled membranous tube coiling around the modiolus: the scala tympani, scala vestibuli, and scala media. The fluid vibrations create a traveling wave along the basilar membrane, in which hair cells generate the electrical signals and pass them to the spiral ganglion cells. **(C)** High-intensity sound waves travel through the cochlear duct, causing damage to three key functional areas: the organ of Corti, the SGNs and the stria vascularis. Hair cells, supporting cells and SGNs go through morphological changes and eventual apoptosis in extreme cases. Loss of tight junctions, malfunction of endothelial cells and surrounding cell types contribute to the BLB disruption. Abbreviations: SV: scala vestibuli; SM: scala media; ST: scala tympani; SCs: supporting cells; OHCs: outer hair cells; IHC: inner hair cell; BLB: blood-labyrinth barrier. Created with BioRender (www.biorender.com).

The vertebrate inner ear is an exquisite design and sensitive sensory organ of serving both the hearing (cochlea) and balance (vestibular) functions. Anatomically, the structures of the ear are divided into three parts: the outer, middle, and inner ear, illustrated in Figure 1A. Sound waves enter the ear canal and travel through the tympanic membrane and the ossicular chain into the fluid-filled cochlea. As shown in Figure 1B, the cochlea is divided into three fluid-filled membranous tubes that coil around the modiolus: the scala tympani, scala vestibuli, and scala media. The sound creates a traveling wave along the basilar membrane, which houses the mechanosensitive hair cells, including three rows of outer hair cells (OHCs) and one row of inner hair cells (IHCs) located in the organ of Corti. OHCs function as a cochlear amplifier and IHCs are responsible for electrical signal transmission to the spiral ganglion neurons (SGNs). Long-term or intense noise exposures beyond safe limits lead to irreversible loss of hair cells, SGNs and other cochlear cell types in the lateral wall, damaging three functional portions of the cochlea (Figure 1C): (1) the organ of Corti for mechanotransduction, (2) the SGNs for electrical signal transmission, and (3) the stria vascularis in charge of the inner ear homeostasis. The noise-related damage to the auditory system and the resulting sensory deafness is termed noise-induced hearing loss (NIHL).

In the last few years, several facts have drawn wide attention, which are also key points that make NIHL different from other sensorineural hearing loss (SNHL). First, we live together with sound or noise in various social and recreational activities. The increasing prevalence of hearing loss is attributed to the audio devices, venues, and events that emit loud music or recreational sounds. According to the World Health Organization, teenagers or young people are the most likely to develop NIHL, with one billion people aged 12 to 35 years at risk 1. The escalating trend in NIHL should prompt changes in the age structure and risk factors. It also encourages personal awareness of safe listening behavior and the need for a new standard for recreational places and events. Second, NIHL has unexpected auditory and non-auditory consequences, which pose an elusive threat to our overall health. Light noise may damage synaptic connections between IHCs and type I SGNs without hair cell loss 2. This is speculated to be connected with “hidden” hearing loss, which is shown by tinnitus, loudness intolerance, or a deficit in hearing under background noise 3. In addition, chronic and repeated exposures activate the body's stress response system and are linked with a higher risk of developing cardiovascular diseases 4 and cognitive issues 5. Recent studies have highlighted the potential role of chronic noise exposure in the development of Alzheimer's disease 6. Third, excitotoxicity to hair cells and neurocytes which involves calcium overload and glutamate neurotoxicity is likely to be one distinct pathophysiology of NIHL as compared to age or drug-related hearing loss. Therefore, current studies of pharmacotherapy with the majority merely focusing on antioxidants should benefit from multiple therapeutic targets.

Currently, the primary methods of prevention of NIHL rely on noise control and hearing protection devices. Hearing aids are the most effective means of restoring auditory perception in patients with mild to moderate hearing loss 7, and cochlear implants are the most effective in patients with severe to profound hearing loss 8. However, it should be noted that hearing protection devices are not suitable for all occasions, and are sometimes not carefully chosen and used in accordance with personal needs. Varied patient outcomes and difficulty in speech perception in noise are typical challenges for cochlear implants 8. Numerous researchers are exploring pharmacologic therapies to prevent, interrupt, or treat NIHL. It is known that cochlear sensory cells in mammals do not possess regeneration ability, making hair cell loss irreversible. However, there is a complex interplay between the cellular and molecular mechanisms of damage and repair, before permanent sensorineural damage sets in. Theoretically, it provides not only a precious therapeutic window for intervention but also a variety of molecular targets against NIHL.

Moderate progress has been achieved in this field. Yet there are no FDA-approved drugs for the treatment of NIHL. The only therapeutic drug option in clinical settings is the off-label use of steroids, either through oral administration or intratympanic injection, due to their known anti-inflammatory effect. Unfortunately, the reported efficacy of steroid therapy is not ideal. This situation has led to numerous of preclinical studies regarding novel drugs and strategies for alleviating hearing impairment. Studies have revealed the highly sophisticated and intertwined cellular and molecular mechanisms underlying the pathogenesis of NIHL. What factors contribute to the pathogenesis of cochlear noise damage? How do various biochemical events, like oxidative stress, inflammation, metabolic damage, Ca^2+^ overload and glutamate neurotoxicity, work together? We have been constantly evolving our understanding, and answers to these questions have become foundations for pharmacological interventions. More and more molecular agents have shown reliable therapeutic efficacy, and some have been tested in clinical trials 9.

The unique isolated anatomical position of the inner ear and the presence of the blood-labyrinth barrier (BLB) make inner ear therapy particularly challenging. Different ways to deliver inner ear drugs, such as systemic and local administration, have varied advantages and challenges. Multiple strategies have already been used to maintain the high permeation of drug molecules through the round window membrane (RWM), but researchers are having difficulty opening the BLB in a systemic delivery. Among them, targeted and responsive drug delivery systems have been highlighted with their current utility and future potential in NIHL therapy.

In this review, we aim to summarize and discuss recent comprehension of the pathological mechanisms underlying NIHL, based on which multiple pharmaceutical explorations have been performed in rodents. The advancing understanding of drug pharmacokinetics across different delivery routes, and the progress in drug delivery systems with targeted and responsive drug delivery capabilities will be analyzed, therefore advancing our understanding of NIHL prevention and treatment. In the end, challenges and opportunities in some critical aspects of NIHL drug therapy are suggested.

## Underlying mechanisms and therapeutic strategies

The auditory system is impacted by two distinct types of acoustic damage: (1) The bioelectrical signal transmission pathway is damaged by acoustic noise. The tympanic membrane, ossicular chain and basement membrane that conduct sound waves can be torn by direct mechanical trauma, often seen in a blast exposure. But in most cases, the hearing loss is rather long-term and progressive, characterized by impairment to the organ of Corti and auditory nerve. They are critical for the mechanotransduction of sound signals and the subsequent transmission of those signals to our brain to be ultimately recognized and understood 10. Moderate-intensity (85 to 96 dB SPL) exposures may lead to temporary threshold shift (TTS) that resolves over days or weeks. However, if the ascending auditory pathway is cut off by the massive loss of hair cells and SGNs after high-intensity noise exposures, patients develop permanent threshold shift (PTS), which is an irreversible hearing impairment. (2) The inner ear homeostasis is disturbed. The chemical and electrical homeostasis in the cochlea is largely maintained by stria vascularis. The unique structure rich in blood vessels serves not only as a gatekeeper for the substance exchanges between the circulation and the inner ear but also as a battery to promote endocochlear potential (EP) formation. Both are essential for normal hair cell function 11. Prier studies have demonstrated significant noise-related abnormalities in EP maintenance, cochlear microcirculation, and integrity of the BLB 12. More detailed structural investigations into stria vascularis revealed tight junction loss between endothelial cells and morphological changes of pericytes and other cell types 13.

**Figure 2:**
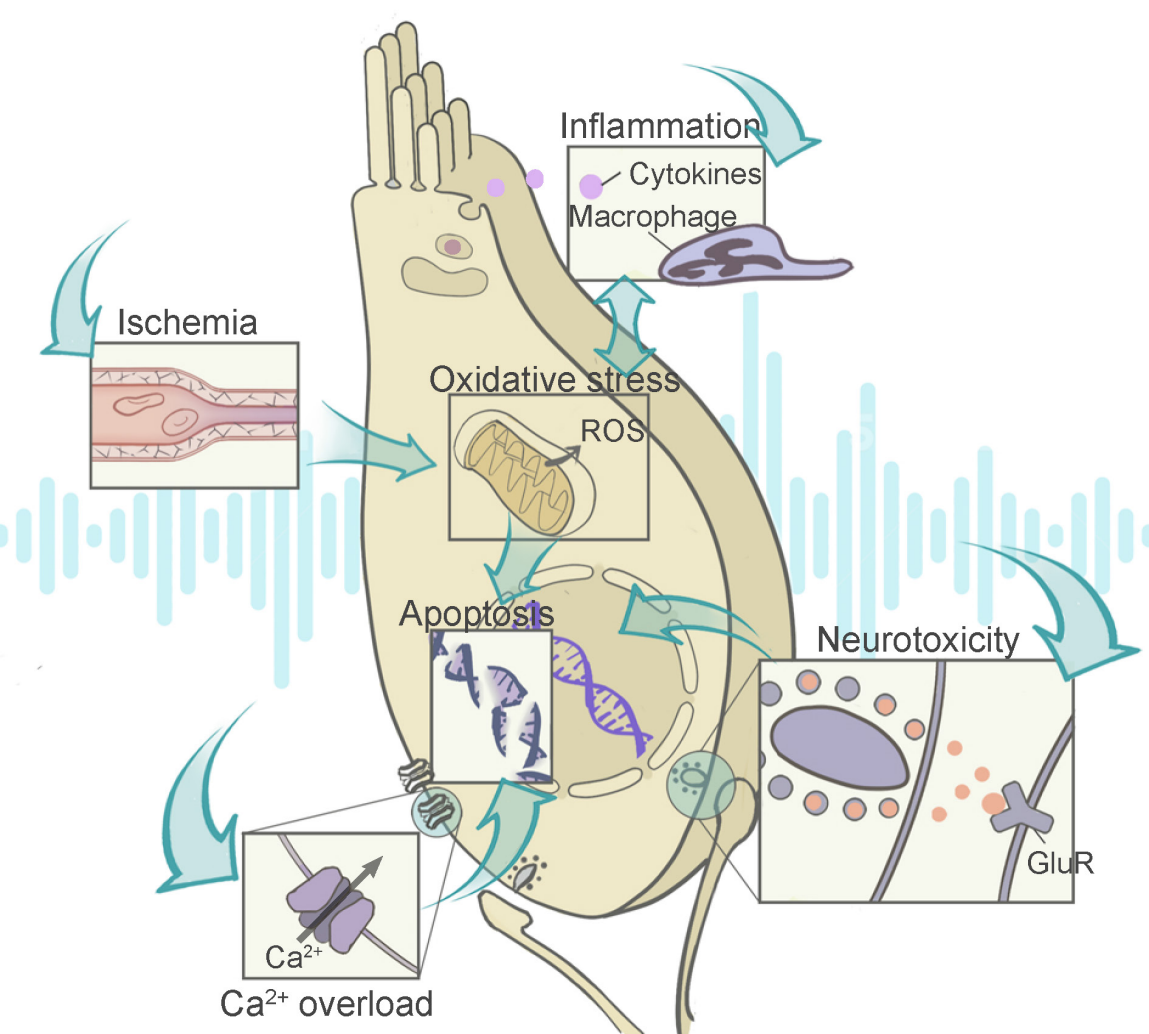
** Illustration of the contributing factors to NIHL and their interactions with each other.** Noise-related disfunction of the stria vascularis leads to ischemia reperfusion injury, which increases the level of ROS in the cochlea. The overproduction of ROS and cochlear inflammation are two biochemical events that promote each other. With excessive calcium influx triggering the glutamate neurotoxicity at the ribbon synapses, both hair cells and the synaptic structures are damaged. Intrinsic or extrinsic pathways of apoptosis are activated by the resulting injuries. Abbreviations: ROS: reactive oxygen species; GluR: glutamate receptor.

As shown in Figure 2, those abnormalities in the auditory system stem from a complex interplay between a number of biochemical events. The reactive oxygen species (ROS) overproduction can be initiated by ischemia reperfusion injury, and mediate a series of signaling pathways involving inflammation and metabolic damage. Some events such as Ca^2+^ overload and glutamate neurotoxicity are pathogenic contributors independent from ROS generation, and they usually prefer certain cell types including hair cells and SGNs. After the initial burst of signal cascade is the subsequent DNA and organelle damages, triggering ultimate cell necrosis or programmed cell death. Therefore, the dominant pathogenic events depend on disease stages and cellular types, which is worth additional consideration during therapy design.

**Figure 3:**
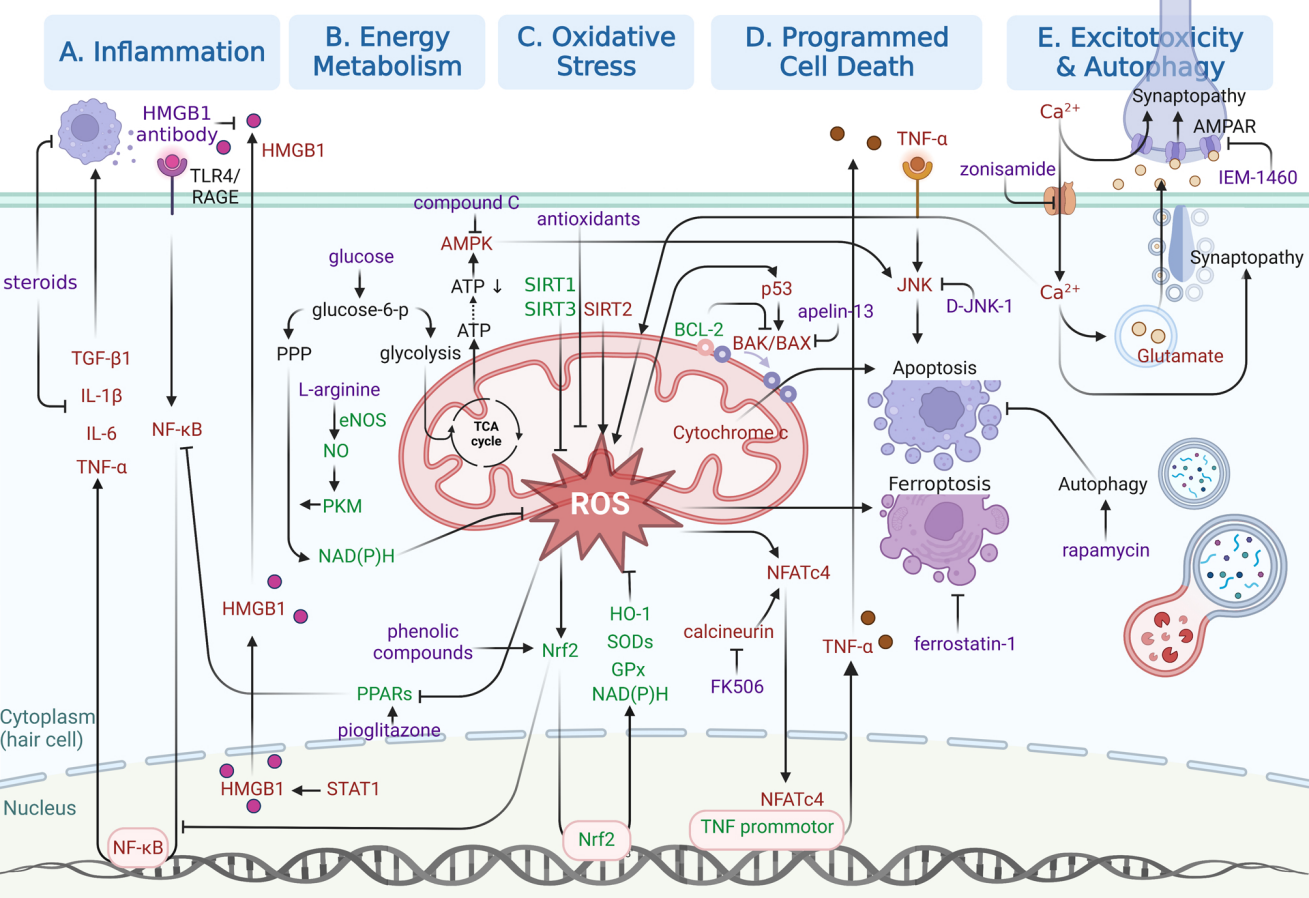
** Illustration of relatively recent findings on molecular pathways underlying NIHL as potential therapeutic opportunities.** Signaling pathways involved in **(A)** inflammation, **(B)** energy metabolism, **(C)** oxidative stress,** (D)** programmed cell death and **(E)** excitotoxicity and autophagy are illustrated in separated regions. Protective responses are shown in green. Adverse cellular events are shown in red. Pharmacological interventions are shown in purple. Created with BioRender (www.biorender.com).

In this section, emphasis will be laid on molecular pathways responsible for NIHL pathogenesis, especially those identified in the last five years. As shown in Figure 3, specific molecular pathways as potential pharmacological targets are highlighted in an attempt to inspire NIHL preclinical drug development. Three kinds of drugs for pharmacological interventions are included in our discussion, including small molecule drugs, biomacromolecules and functional oligonucleotides.

### Oxidative stress and antioxidative therapies

Following PTS-producing noise, ROS overproduction took place right after the insult, reached a maximum at 7 to 10 days and then declined, indicating a ROS accumulating stage for potential therapeutic intervention 14. There is no sufficient evidence that oxidative stress is involved in the late stage of NIHL. The overproduction of ROS originates from multiple sources. Intracellular calcium overload, stimulated by noise exposure, leads to loss of mitochondrial membrane potential and leakage of ROS into the cytoplasm 15. Upregulation of NADPH oxidase, reduced cochlear blood flow and ischemic/reperfusion damage all give rise to initial ROS production 16-18. Numerous downstream factors in ROS signaling mediating inflammation or apoptosis further exacerbate ROS generation in a vicious cycle. For example, BAX and BAD are pro-apoptotic proteins that decrease the mitochondrial potential and cause more ROS to be released 19. When excessive ROS generation is far beyond what the antioxidant system can neutralize, it can lead to morphological nuclear changes 20, DNA damage 21, lipid peroxidation 22, and ultimately necrosis or apoptosis. Furthermore, it has been demonstrated to directly induce apoptosis through the MAPK-c-JNK pathway 23.

Since ROS generation is a significant contributor to noise-induced cochlear damage, a typical therapeutic intervention involves neutralizing or suppressing its generation through antioxidant supplements. There are some intrinsic antioxidant molecules with *in vivo* production (e.g., glutathione 24, N-acetylcysteine(NAC) 25, and ebselen 26). Others are exogeneous antioxidant supplements including resveratrol 27, d-methionine 28,29, vitamin C 30 and coenzyme Q10 31. NAC, which is a precursor of glutathione, is the most studied antioxidant to reduce acoustic damage in both rodent models and human studies 25,32-34. The efficacy of this drug varies among studies, but it is still the only studied molecule that has entered a phase 3 clinical trial 35. Herbal products from traditional medicine are one of the main sources of drug discovery. Several herbal-derived compounds (e.g., resveratrol 27, curcumin 36, and baicalein 37) have been investigated for their antioxidant capability in NIHL animal models. Notably, many of those herbal-derived compounds are reported to have multiple targets simultaneously including oxidative stress, inflammation, apoptosis and neurodegeneration 38, but the details of their mechanisms are often not fully elucidated. For other concerns, bioavailability differs among antioxidants, and some antioxidants react with only a few forms of free radicals, or may throw innate stress-protective response out of balance 39.

Another approach is to start by revealing ROS signaling pathways involved in the pathogenesis of NIHL, either protective or devastating. Drugs or functional oligonucleotides targeting those pathways are then investigated (Figure 3C). ROS are essential for maintaining normal cell homeostasis. They function as critical modulators of stress-protective response after a mild noise insult, trying to restore reducing equivalents. Sound conditioning is a great example of using mild exposures to prepare innate protective systems for more severe stress 40. Only when noise exposure has reached a damaging level does ROS trigger a down-stream cascade of oxidative stress and other adverse cellular events. In those cases, the devastating consequences are more likely the result of an overwhelming oxidative stress over stress-protective response. It is difficult to determine which strategy performs better, motivating the innate protective mechanism of ROS, or suppressing pathways to adverse cellular events. NRF2/HO1 pathway was thought to be a major part of the defense system against NIHL 41. In response to oxidative stress, nuclear factor erythroid2-related factor 2 (NRF2) transcriptionally upregulates the expression of innate antioxidants (heme oxygenase-1 (HO-1), Superoxide dismutase (SOD) and glutathione peroxidase (GPx)), increases reducing equivalents (NAD(P)H), and interferes with inflammatory signaling pathways 42. Recently, activating transcription factor (ATF), a basic leucine zipper transcription factor, has been shown to be a key therapeutic target for modulating NRF2 and cellular defense system against NIHL 43. Yang's group further explored the avenue by revealing the protective role of eNOS-NO-PKM2 in glucose metabolism against NIHL 44. Endothelial nitric oxide synthases (eNOS) and nitric oxide (NO) increased in noise-exposed cochlea, directing glucose metabolism to pentose phosphate pathway with more reducing equivalent produced by pyruvate kinase M2 (PKM2). L-arginine, which is a precursor of NO, was found to be effective, and more future therapies modulating NO or glucose metabolism possess great potential. Furthermore, sirtuin (SIRT) proteins, a group of NAD-dependent deacetylase, have been focused in recent years. Unlike the pathways mentioned above, there is a diversity in the functions of those proteins. Studies revealed that SIRT2 pathways cause mitochondrial oxidative stress and apoptosis 45, but SIRT1 and SIRT3 stabilize the mitochondrial function and attenuate oxidative stress and apoptosis in animal models 46-48. So far, the therapeutic value of modulating SIRTs in NIHL models has been proved 45,47,48, but the details of their role in cochlea after noise exposure: the upstream modulators and downstream effectors have not been fully elucidated. More in-depth mechanism and *in vivo* drug investigations deserve consideration.

It is important to note that some factors hampered the therapeutic applications in modulating ROS signaling pathways. The target molecule may possess multiple biological functions, or may be involved in multiple analogues. Additionally, some regimes lack specificity for their targets, which might lead to off-target effects 49.

### Inflammation and anti-inflammatory therapies

Robust cochlear inflammation occurs almost immediately in response to acoustic trauma, which can be characterized by recruitment of inflammatory cells, as well as generation of inflammatory mediators in the cochlea 50. For unknown reasons, neutrophils do not participate in this response. The primary mediators of this process are bone marrow-derived monocytes and macrophages 51. Monocytes from the blood stream are recruited by chemokines, infiltrate through venules in the lateral wall within 2 days and then subsequently transform into macrophages upon entering the inner ear 50. Furthermore, there are abundant resident macrophages present in the spiral ligament, which experience activation and rapid proliferation. It appears that systemic depletion of macrophages is protective for acute acoustic damage, and therefore it seems that the macrophage activities are generally harmful in NIHL 52. However, the study did not classify macrophages or monocytes because the role of those cells in the cochlear immune response can be complicated and likely depends on the stage of the disease. When noise insult upsets the balance of dynamic equilibrium, they secrete a lot of pro-inflammatory cytokines, such as tumor necrosis factor-alpha (TNF-α), interleukin-1beta (IL-1β), and interleukin-6 (IL-6) 53,54. After the initial burst of inflammation is over, cell debris after apoptosis or necrosis is phagocytosed by the infiltrated and resident macrophages 55. Intriguingly, a novel function of those macrophages was reported in promoting IHC ribbon synaptic repair 56. Overall, multiple factors govern macrophage dynamics and transformation of subtypes. With more explicit knowledge, we might be able to direct infiltration and other biological activities of monocytes and macrophages, or mediate the status of macrophage subtypes in the inner ear. The potential of those strategies in treating NIHL has not been fully investigated yet.

From another perspective, the regulation of inflammatory and immune response is based on intricate biological processes that involve both immune cells and non-immune cells (Figure 3A). The generation and signaling of cytokines and chemokines in those cells are key targets for some medications. Preserving hearing function and cochlear morphology before and after noise exposure can be achieved by inhibiting transforming growth factor beta 1 (TGF-β1) signaling with the blocking peptides 57. High mobility group box 1 (HMGB1) is another extracellular mediator of inflammatory response, and is an extensively explored damage-associated molecular pattern 58. HMGB1 acts as an indicator of cell damage, which accelerates inflammation in neighbor cells and stimulates professional scavengers. Its direct binding to the toll-like receptor 4 (TLR4) initiates NIHL via the NF-κB and MAPK signaling pathways 59. Translocation of HMGB1 from the nucleus to the cytosol is the prerequisite for its intracellular function and subsequent cellular release. Any contributors to HMGB1 acetylation (e.g., SIRT1) were speculated to manipulate the process and hold promises as future therapeutic targets 60,61. The existing literatures have described successful applications of HMGB1-neutralizing antibodies for the precise blocking of extracellular HMGB1 functions 60,62. It is suggested that the early development of inflammation involves a crosstalk with ROS signaling, and that targeting their interplay might cut off the progression of NIHL. Researchers found peroxisome proliferators-activated receptors (PPARs), a family of three ligand-regulated transcription factors, have critical role in regulating oxidative stress/inflammatory interplay in the NIHL 63. Oxidative stress suppresses the expression of PPARs, which in turn facilitates inflammation by preventing negative interactions with NF-κB. The same research group demonstrated the therapeutic potential of targeting PPARs by the local delivery of pioglitazone, a PPARγ agonist, for NIHL treatment 64.

Traditionally, corticosteroids (e.g., prednisolone, hydrocortisone, and dexamethasone) have been extensively evaluated in NIHL treatment 65-69. It is suggested that the steroid action in the cochlea extends far beyond anti-inflammation 70, but heterogeneity in treatment outcomes and side effects due to broad therapeutic targets hinder their application. Nevertheless, the use of steroids enlightened the exploration of unknown mechanisms underlying the progression of NIHL. Shih et al. showed that dexamethasone considerately suppressed the upregulation of HMGB1 in the spiral ligament and ameliorated cochlear and hearing impairments 71. To our knowledge, it is the first study to show that HMGB1 is involved in noise-induced cochlear inflammation. Nelson et al. conducted a comparative analysis of steroid-responsive genes in specific cochlear cell types using published transcriptome and single-cell RNA datasets 70. The research identifies differentially expressed steroid-responsive genes and pathways as potential drug targets, particularly for specific cell types. The group also utilized gene target databases to identify existing FDA-approved drugs, which may be repurposed for NIHL treatment 72.

### Energy metabolism dysfunction and related therapies

The role of the cochlear lateral wall involved in the disease pathogenesis probably has been underestimated. It is well known that extensive noise diminishes normal cochlear blood flow, inducing ischemia/reperfusion damage and a surge of oxidative stress 73. Studies have revealed elevated vasoactive gene expression and an accumulation of vasoconstrictor in the cochlear lateral wall 18,73. The overwhelming vasoconstriction factors are speculated to originate from damages to fibrocytes or pericytes in the cochlea, which is highly responsible for orchestrating the cochlear capillary system 74.

Magnesium supplements have been suggested as a treatment for NIHL for a considerable period of time 75-77. As a vasodilator, magnesium is also known for a variety of pharmacological properties, such as blocking NMDA receptor, attenuating glutamate overproduction, and reducing calcium influx into hair cells 78. Some vasodilators used in cardiovascular diseases have a long history of clinical application and a well-established safety profile. Among them, milrinone, a PDE III inhibitor, and papaverine, a direct-acting vasodilator, have received preclinical investigations in rodent animals and shown protective effects for hearing impairment and hair cell loss 79,80.

Hypoxia and energy depletion escalate the metabolic dilemma in noise induced cochlear damage (Figure 3B). The oxidation defense system is also energy-consuming. Transient ATP depletion was reported following acoustic damage due to increased mitochondrial aerobic respiration 17. However, a glucose supplement partially restored the ATP level and enhanced antioxidant defense by a single dose injection shortly before noise exposure 81. The details of how glucose metabolism works in NIHL are still unclear. And the study raised the question of whether a high-sugar diet enhance cochlear tolerance to acoustic damage. Furthermore, the ATP depletion exhibits downstream effect on adenosine monophosphate-activated protein kinase (AMPK). It is a critical energy modulator sensitive to AMP/ATP increase and upregulate energy generation 82. Moderate activation of AMPK boosts energy supply, which might be protective against NIHL. By sound conditioning using low-intensity noise, an innate causality was implied between activated AMPK and reduced hearing loss or synapse damage 83. However, excessive and sustained activation of AMPKα signaling is responsible for subsequent c-Jun N-terminal kinase (JNK) mediated apoptosis of hair cells 84. Studies showed that inhibition of AMPK or its upstream AMPK kinase liver kinase B1 (LKB1) significantly reduced the loss of OHCs and synaptopathy 85. Those findings also indicated that AMPK signaling plays a critical role in cochlear synaptic disorders.

### Autophagy and anti-autophagic therapies

Recently, it has been thought that autophagy is involved in the development of SNHL including hearing loss originated from noise exposures, ototoxic drugs and aging 86. In response to multiple stress conditions, abnormal intracellular substances or organelles undergo certain trafficking and degradation processes, providing extra energy or maintaining cellular homeostasis 87. However, limited studies have focused on the role of autophagy in the defense system against NIHL. Yuan et al. reported that increasing the autophagy level protected against noise-induced hair cell loss, while suppressing it exacerbated oxidative stress and noise damage. All of these effects were achieved using pharmacological or siRNA tools (Figure 3E) 88. Apart from its pro-survival effect, autophagy is known as the inducer of cell apoptosis in many diseases 89, but research data did not support a pro-apoptotic role of autophagy in NIHL since the autophagy marker was found significantly elevated after a mild noise exposure but only slightly increased in more severe noise damages 88. Further complementary researches, based on phenotyping genetically modified animal models, are in critical need because pharmacological inhibitors and activators target other cellular pathways as well. It seemed that activation of autophagy alone yielded only minimal hearing and morphological improvements, but synergistic action with other targeted pathways achieved more efficient protective effects 90.

### Calcium-induced glutamate neurotoxicity and related therapies

A series of innate intracellular mechanisms are responsible for signal processing between IHCs and type I SGNs, including the calcium-triggered glutamate release from IHCs, glutamate transmission and Ca^2+^ influx into the postsynaptic terminals (Figure 3E) 91. Those areas are extremely vulnerable to noise-induced excitotoxicity, leading to hair cell loss and synaptopathy. Specifically, the damaging effect of noise on synapses has been associated with noise-induced hidden hearing loss 3.

Preventing the damage at first place and promoting synaptic recovery are two ways to diminish the harm of noise-induced cochlear excitotoxicity. Voltage gated calcium channel (VGCC) blockers have been evaluated considering that the calcium influx into overstimulated hair cells via VGCCs is the initial step towards excitotoxicity 92. Their protective effect was evident, especially in preventing hair cell loss, but which type of VGCCs, T-, or L-type, that is most responsible for the calcium overload in hair cells remains controversial 93,94. Although Ca^2+^ influx is considered to be a key mediator of synaptic trauma, there has been no observed improvement in synaptopathy by using VGCC blockers up to now, presumably because postsynaptic calcium influx is not mainly driven by VGCCs, but calcium permeable glutamate receptors (GluRs) instead 95. Among the various GluR types, AMPARs, NMDARs, and kainite-Rs, NMDARs have a major role in Ca^2+^ influx in the brain 96, but new evidence suggested that AMPARs are major contributors to Ca^2+^ influx and synaptic excitotoxicity in the cochlea 95. By selectively blocking the Ca^2+^-permeable AMPARs via chronic intracochlear delivery of IEM-1460, both the synaptic trauma and the reduction of cochlear nerve activity in response to sound were significantly attenuated 97. Indeed, the surface Ca^2+^-permeable AMPARs is in dynamic changes with constant endocytosis and replenishment 98. Another therapeutic approach may be down regulating the membrane AMPARs by facilitating their internalization 99.

The degree of recovery in synapse number and function may vary in different settings of noise exposure or animal species and strains 100. In order to promote the synapse recovery, exogenous application of trophic factors, including neurotrophin-3 (NT-3) and brain-derived neurotrophic factor (BDNF), was probably the most evaluated medication. Studies have demonstrated that both exogenous supplementation and genetic upregulation of NT-3 significantly enhanced synaptic regeneration following noise-induced synaptopathy 101,102. In recent years, other approaches have been pursued to better harness synaptic regeneration. Manickam et al. reported that cochlear resident macrophages were necessary and sufficient for the recovery of noise-induced synaptopathy 56. It should be a recovery mechanism parallel to the action of neurotrophins because neurotrophins in cochlea were expressed by hair cells and supporting cells, not macrophages. Another study by Kim et al. showed the unexpected role of glutamate release in synapse regeneration after noise exposure, indicating that the regeneration may require normal postsynaptic Ca^2+^ influx 103. These findings revealed a complex network of signaling pathways involving multiple cell types, which may serve as therapeutic targets.

### Programmed cell death and related therapies

The progress of programmed cell death is mediated by the activation of multiple cell death pathways described in the preceding sections. The higher the noise level, the greater the difficulty in achieving meaningful physiological protection through intervention in the downstream cascades. Mostly evaluated and targeted are ROS-mediated and inflammation-mediated hair cell apoptosis, realized by the sequential action of caspases (Figure 3D) 104. However, direct inhibition of caspases was recognized to shift OHC death to necrosis 84. Over the past decade, researchers have been seeking anti-apoptotic and pro-apoptotic regulators in the development of apoptosis. There are two different but interconnected pathways. In extrinsic pathway involving inflammation and cytokines, MAPK-c-JNK pathway has a crucial role mediating the activation of apoptosis. Synthetic JNK blocking ligand (D-JNK-1) attenuated noise trauma with intratympanic administration in guinea pigs, and different formulations based on mini pump or hydrogels have been developed 105-107. In intrinsic pathway modulated by the mitochondrial and oxidative stress, mitochondrial-related apoptosis is mediated by Bax/Bcl2 balance. Upregulation of Bax/Bcl2 ratio induces the release of pro-apoptotic factors like cytochrome C from the mitochondrial intermembrane space, triggering the subsequent activation of caspase9 and caspase3 108. A recent study showed that apelin-13, a neuropeptide, had strong anti-apoptotic effects. This effect was linked to a change in the Bax/Bcl2 balance and an increase in SIRT1 in the mitochondrial-related apoptotic pathway 48. Additionally, the nuclear factor of activated T cells (NFAT) protein family was found to be a critical component in the crosstalk of ROS signaling and TNF generation. The expression and function of NFATc4, specifically, was found in cochlear hair cells 109. When activated by ROS or calcineurin, they translocate to the nucleus and upregulate the expression of TNF and TNF-mediated hair cell apoptosis. The calcineurin inhibitor FK506 attenuated noise-induced hearing loss and OHC loss, but the protective effect diminished greatly for aminoglycoside-related ototoxicity 90,109, indicating different roles of calcineurin/NFAT signaling in the pathophysiology of the two diseases.

It is likely that not only apoptosis, but also several types of programmed cell death, including necroptosis, pyroptosis ferroptosis and cuproptosis, are triggered by noise stimuli 110. Those critical modulators or crosstalk molecules within the signaling network provide new avenues for NIHL treatment. But supporting evidence for this assumption is lacking in the scenario of NIHL. The first breakthrough comes from a recent study that showed that ferrostatin-1 (Fer-1), a well-known ferroptosis inhibitor, protects against NIHL. This shows that ferroptosis is involved 111.

## Translational dilemma from animals to human

We have emphasized the diversity of cellular pathways that are involved in the pathogenesis of NIHL, hoping to bring inspiration to new medications for the prevention or the treatment of the disease. We wonder if there is some sort of 'command center' that controls how cells react to stress. Since there is no clue, combining different drugs to have synergistic effects or using drug that has multiple actions has proved to be highly successful 90,112. There are concerns that excessive activation or suppression of certain pathways may disturb the balance of innate defense system 113.

**Figure 4:**
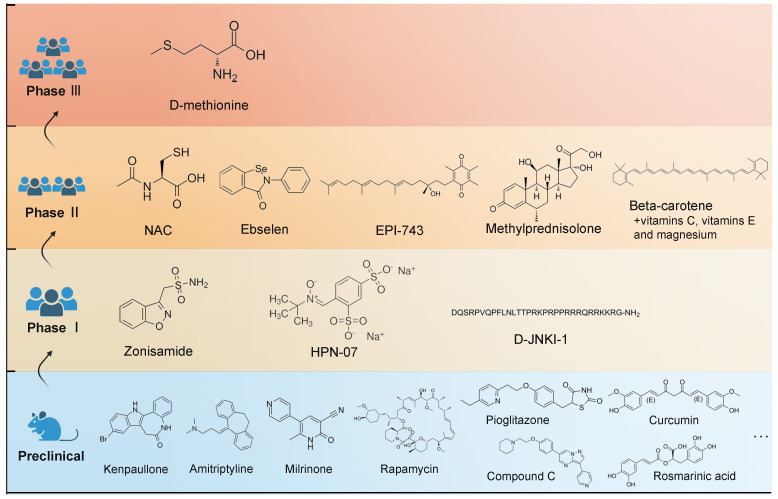
** Molecular structures of the clinical candidates for NIHL therapy.** A non-exhaustive list of evaluated drugs in preclinical studies is illustrated in the blue region. Drugs that have entered clinical trials are illustrated in separated regions according to the stage of clinical trials they are in.

With new inspirations and strategies, a broad range of potential medications have demonstrated therapeutic values in animal studies, which has been reviewed by Isabel's group 1. However, a limited number of them have been subjected to clinical testing (Figure 4). The database of the ClinicalTrials.gov, which was searched on April 10, 2023, currently contains 12 registered clinical studies exclusively focusing on NIHL pharmacologic treatment, including six completed clinical trials. The antioxidant activities of N-acetylcysteine have been identified 114, but a combination therapy of antioxidant vitamins and the mineral magnesium fail to prevent temporary threshold shift 115. Notably, ebselen showed significant efficacy in alleviating a temporary threshold shift in a phase 2 trial 116. A phase 2b study of FX-322, a promising drug aiming to restore hearing by hair cell regeneration, was recently completed with a disappointing result. Overall, there were setbacks, which might be attributed to the weaknesses in the study designs or perhaps, in the drug delivery approaches.

There are some limitations and haunting questions in a reexamination of the development of pharmacological interventions of NIHL: (1) Most studies do not distinguish between noise types. A representative noise design in an experimental model is a narrowband or broadband noise with constant power spectral density at a constant sound pressure level for a few hours 1. However, the noise in the real world is a mixture of various patterns. For example, the temporal characteristics of noise. In fact, many people develop NIHL from moderate and repeated exposures lasting probably for years rather than from acute ones, which has been given limited attention when designing preclinical animal models. (2) The therapeutic window for treatment after noise is yet unknown. It might depend on the drug and the time course in which the targeted pathway was involved. It was found that ROS was generated immediately after the insult and reached a maximum of 7 to 10 days 14. According to it, maximal effectiveness of an antioxidant therapy was achieved when the therapy was initiated within 24 h and extended for 10 days after noise exposure 117. But supporting evidence for the therapeutic window of other drug types is scarce. (3) Drug delivery to the inner ear is a challenging task in itself. The pharmacokinetics of numerous drugs in the inner ear are indeterminate, posing challenges in maintaining effective drug concentration in this difficult-to-reach sensory organ. It is a dynamically evolving research field that we will discuss in the following sections.

### Improving inner ear drug delivery efficiency across biological barriers

**Figure 5:**
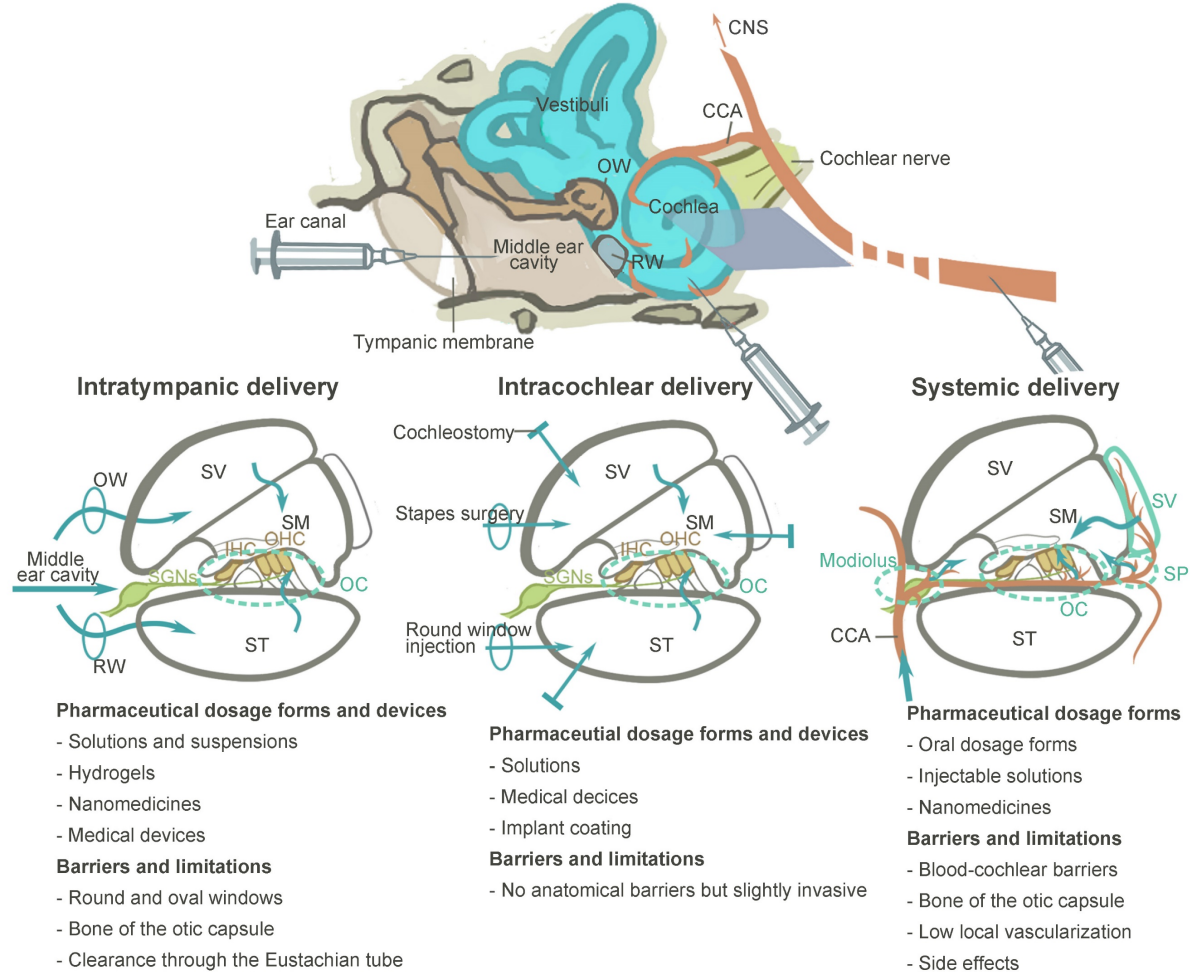
** Summary of different drug delivery routes to the inner ear.** Abbreviations: CCA: common cochlear artery; CNS: central neural system; IHCs: inner hair cells; OC: organ of Corti; OHCs: outer hair cells; OW: oval window; RW: round window; SM: scala media; SP: spiral prominence; ST: scala tympani; SV: scala vestibuli.

Pharmacological interventions with physiologically meaningful protection against NIHL are complicated by the fact that the drug has to be delivered to the inner ear across multiple biological barriers. In fact, it is a common challenge facing all SNHL therapies. Approaches to the inner ear can be categorized into two groups: systemic delivery and local delivery (Figure 5). Systemic delivery must overcome the BLB, and local delivery is hindered by the isolated anatomical position and tissue barriers. For achieving efficacy, several key design principles were introduced for both classes of delivery approaches.

### Systemic administration across the blood-labyrinth barrier

Systemic administration used to have a dominant role in clinical studies and clinical practices of NIHL treatment, due to its feasible and non-invasive nature (Figure 5). It is cost-effective when compared to intratympanic delivery because it does not require a repeated visit to professional healthcare facility and an experienced otolaryngologist to perform the whole procedure which include otoscopy, local anesthesia, administration and the following resting time 118. Although the outcomes varied, several orally administered regimes, mostly corticosteroid therapy, have shown overall improvement in audiometric results and clinical outcomes 119-121.

However, the presence of the BLB casts a shadow on the theoretical basis of this delivery approach. Several studies suggested that drug passage from the bloodstream into the inner ear fluid is scarce (around 0.000005% for methylprednisolone) 122. A human study showed that the concentration of dexamethasone in the inner ear after systemic administration was about 88 times lower than that after intratympanic injection 123. The difficulty of obtaining appropriate drug concentrations within the therapeutic window also poses a great danger of significant adverse effects on other organs.

**Figure 6:**
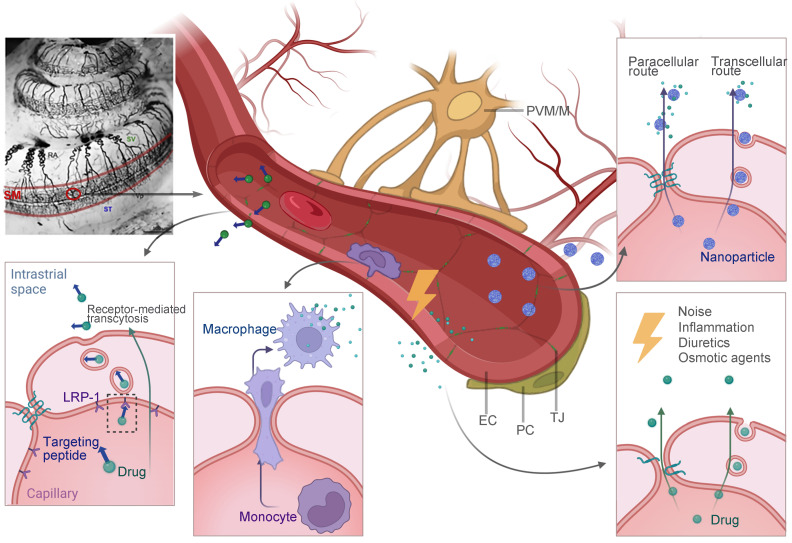
** Potential future strategies for drug delivery across the BLB that have been discussed in this review**. Multiple strategies including receptor-mediated transcytosis, cell-mediated drug delivery, nanoparticle-facilitated drug delivery and exploiting certain conditions to open the barrier are illustrated. The image of cochlear blood supply by ink perfusion was reproduced under the terms of the CC-BY Creative Commons Attribution 4.0 International License (https://creativecommons.org/licenses/by/4.0/) 11. Copyright 2018, the authors, published by Elsevier. Abbreviations: EC: endothelial cells; LRP-1: low-density lipoprotein receptor-related protein 1; NPs: nanoparticles; PC: pericyte; PVM/M: perivascular resident macrophage type-melanocyte; TJ: tight junction; SM: scala media; ST: scala tympani; SV: scala vestibuli. Created with BioRender (www.biorender.com).

BLB is a functional barrier that controls substance exchange between the systemic vascular system and the inner ear fluid. In the inner ear, regions with blood vessel distributions could be part of the BBB, such as the modiolus, the spiral prominence, the organ of Corti and most importantly, the stria vascularis 124. As shown in Figure 6, the intrastrial space contains a complex branching system of capillaries, together with pericytes and perivascular-resident macrophage-like melanocytes (PVM/Ms) 13. On the medial surface of the stria vascularis is a layer of tight junction-coupled marginal cells that separate it from the endolymph in the scala media. Abundant publications have suggested that pericytes and PVM/Ms indirectly maintain the barrier integrity 13,125, while transcellular or paracellular transport is directly limited by two layers of junction-coupled cells: the endothelial cells of the capillaries and the marginal cells 124.

Despite this, there are opportunities for drug delivery across the BLB (Figure 6). The majority of the advanced strategies in this review are still in the early stages of development. To begin with, substances with low molecular weight are basically favored for the passage through the BLB, but there is a selectivity for certain small molecules such as ototoxic drugs. For example, Gentamicin was found to enter the inner ear via an endocytic transporter, megalin (LRP-2), expressed in the marginal cells of stria vascularis 126. Although the idea of utilizing the innate biological transport mechanisms to cross the BLB for drug delivery is still in its infancy, it could be facilitated in the near future. One recent study found that the low-density lipoprotein receptor-related protein 1 (LRP-1) on the BLB can be a potential target for shuttling therapeutics across this barrier 127, and its therapeutic value in facilitating the NIHL treatment has not been testified yet. Another approach to be explored in this field is cell-mediated drug delivery. Based on the observation that circulating macrophages and monocytes actively migrate into the inner ear under both physiological and stress conditions 50,128, they could be engineered for therapeutic purposes. Secondly, nanoparticulate systems have been widely investigated as drug delivery platforms for central nervous system (CNS) disorders across the blood-brain barrier 129. Although non-targeted nanoparticles are generally considered less permeable to biological barriers, it was reported that a zeolitic imidazolate framework-based nano system loaded with steroids efficiently accumulated in the inner ear, and protected the cochlea from noise damage 130. The research and subsequent investigations have been unsuccessful in elucidating the precise mechanism of nanostructures passing through the BLB 131. Advanced technologies, like the surface decoration of peptides that target or penetrate, may further improve the drug delivery efficiency. Lastly, certain conditions were reported to improve the barrier permeability, including inflammation 132, diuretics 133, and osmotic agents 134, which provide the possibility for drug entry theoretically. An animal model of drug-induced hearing loss showed that a combination of antioxidants with mannitol, which is a diuretic medication, improved the otoprotective effect of the antioxidative therapy 135. It shows that this therapeutic strategy has a lot of potential in NIHL research.

### Local administration across the tissue barrier

Considering the limitations of the traditional drug delivery routes, there has been an increasing interest in local administration, which bypasses the vascular systems and the BLB to reach the inner ear more direct and efficiently. Local administration is represented by two basic approaches: intratympanic and intracochlear approaches (Figure 5).

#### Intratympanic delivery

Intratympanic drug delivery is nowadays the mainstream delivery route in preclinical animal studies of NIHL, and is extending its applications to clinical trials. There are two biological barriers including the tympanic membrane (TM) and the RWM before reaching the inner ear 11. The TM separates the outer and the middle ear. Within the tympanic cavity, the ossicular chain is vital for sound conduction and treatment-related trauma should be avoided. The eustachian tube, leading from the middle ear to the throat, would serve to clear drug formulations delivered in the middle ear 136. The last barrier separating the middle ear from the inner ear fluid is the RWM or oval window (OW) 137.

**Figure 7:**
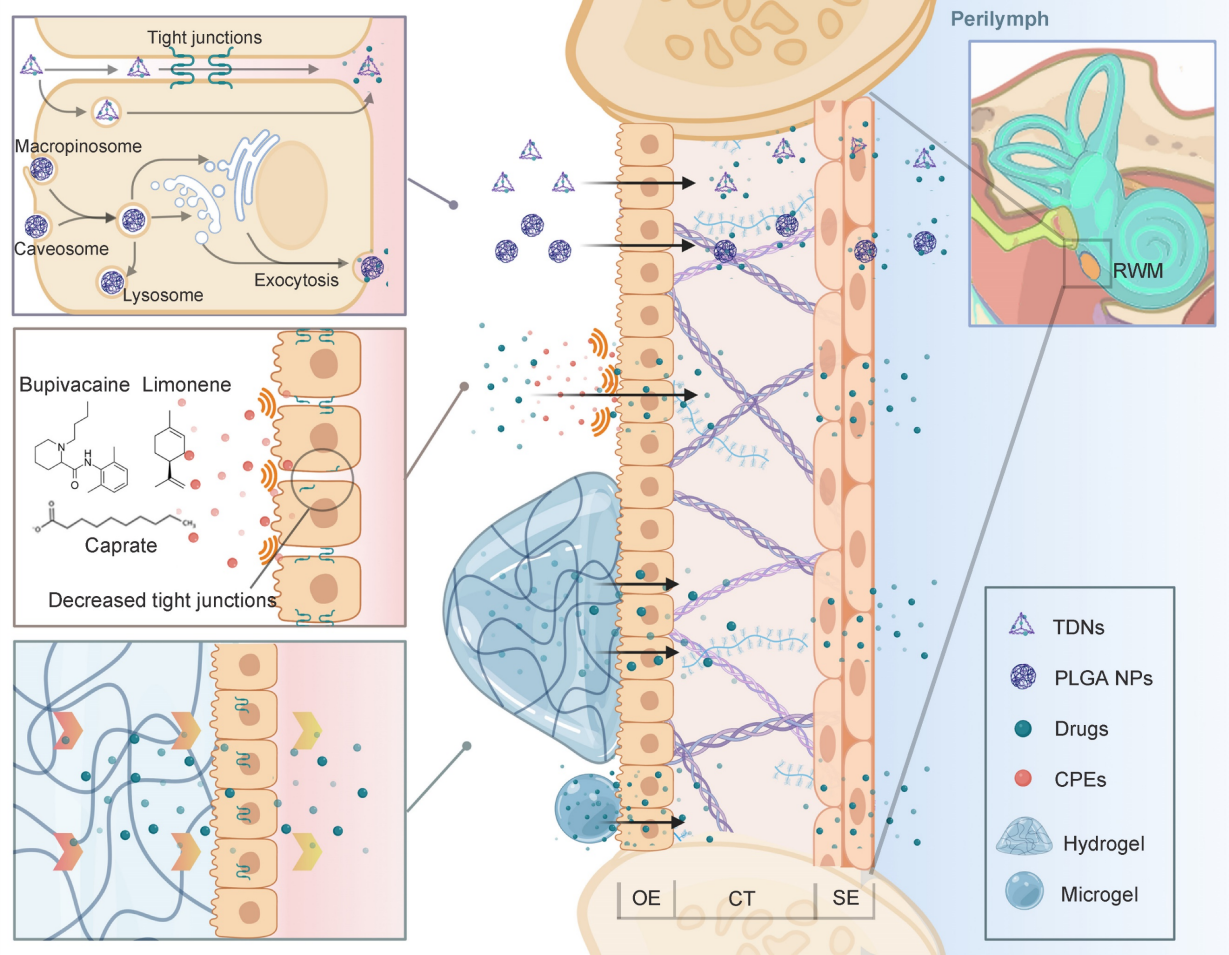
** Advanced strategies for intratympanic drug delivery across the RWM that have been discussed in this review.** Nanoparticulate systems can penetrate the tissue barrier via both paracellular and transcellular pathways. CPEs can be used to decrease the tight junctions to improve drug penetration. Gelling systems maintain close attachment to the RWM for prolonged drug delivery. Abbreviations: CPE: chemical permeation enhancers; CT: connective tissue; OE: outer epithelium; RWM: round window membrane; SE: squamous epithelium; TDNs: tetrahedral DNA nanostructures. Created with BioRender (www.biorender.com).

Since the TM is much more impermeable than the RWM, a direct injection through TM into the tympanic cavity is usually involved 138. Ultimately, the RWM becomes the main challenge of drug diffusion into the inner ear, considering that the OW is partly occluded by the stapes 139,140. The RWM is semi-permeable and the drug diffusion through RWM bears some resemblance to that through skin. To sum up, the drug delivery involves two general principles: (1) maintaining close contact with the RWM for the longest possible period, and (2) improving the transport efficiency through the RWM (Figure 7).

Biocompatible materials such as hydrogels can retain drug solution or drug loading nanoparticles in its polymer structures. When injected into the tympanic cavity or placed on the RWM, the gels with their high viscosity ensure the longest drug retention, and minimize the drug clearance from the eustachian tube 141. Based on different polymer compositions, gels can have sheared thinning behavior or finely tuned thermosensitivity, which means they can be easily applied by a syringe or gel quickly at body temperature. One tricky problem is that, in clinical researches, placing the gel precisely into the round window niche is challenging during an intratympanic injection. While in most NIHL animal studies, the gels were surgically placed on the RWM after opening the temporal bone. This detail should be noted in future NIHL translational researches. Besides, bulk hydrogels can induce conductive hearing impairment due to their large volume 142. Microgels, which are basically micro-scale hydrogels, also possess adhesion ability but will preserve the normal acoustic wave transmission 143.

To improve the transport efficiency through the RWM, advanced drug delivery systems including nanostructures have intensively been applied for inner ear drug delivery. For one thing, nanocarriers improve the drug bioavailability by controlled release and higher drug stability 144. For another, PLGA nanoparticles as an example, were found to be actively transported within the RWM via multiple mechanisms of cell endocytosis and exocytosis 145. Our research group has designed an inner ear drug delivery system based on tetrahedral DNA nanostructures (TDNs) 146. The TDNs rapidly penetrated the RWM 0.5 h after administration and delivered epigallocatechin gallate for hearing protection in the NIHL model. It is supposed that TDNs can penetrate the tissue barrier via both paracellular and transcellular pathways 147. This field of research has been propelled by engineering of intelligent drug delivery systems 144. Beyond that, the permeability of the membrane can be improved by pre- or cotreatment with chemical permeation enhancers (CPEs). For example, Jeong et al. attempted to modulate the tight junctions of the RWM using a medium chain fatty acid, caprate. The co-treatment of caprate and dexamethasone led to enhanced drug diffusion and significantly relieved noise-induced hearing loss 148. Other CPEs, including bupivacaine, limonene and sodium dodecyl sulfate, or certain biomaterials such as chitosan nanoparticles have great potential to facilitate the paracellular transport pathway across the barrier in future NIHL treatment 149,150.

#### Intracochlear delivery

This delivery approach provides direct access to the inner ear, without the drug bioavailability being affected by multiple biological barriers. For intracochlear delivery, drugs can be injected with a fine needle through the RWM or by a cochleostomy 151,152. However, the overall application of intracochlear delivery in this field is restrained by its invasive nature. The volume and pressure changes induced by the injection or efflux of perilymph from the opening can damage the fragile sensory epithelium of the cochlea 153,154.

To diminish the harm, a microcatheter connected to an osmotic or infusion pump enables long-term, ultra-low and highly controlled delivery rate 155. Due to its advantage in allowing application of a precisely designed drug concentration bypassing biological barriers, it is a great tool for evaluating drug efficacy in basic research. Malfeld et al. developed a NIHL animal model with an osmotic pump-based system for evaluation of preventive drug effects 156,157. The current pump- and catheter-based intracochlear delivery system is still too traumatic for NIHL therapies in human patients. To maintain fluid volume, an infuse-withdraw delivery pattern that mixes drugs into perilymph has been developed 158,159. The administration of BDNF can, in part, temporarily ameliorate the hearing impairment caused by the chronic intracochlear delivery devices 160.

### Targeted and responsive drug delivery strategies in NIHL

For the last decades, drug delivery systems have been increasingly utilized in both systemic and intratympanic routes to improve the therapeutic outcomes of NIHL treatments (Table 1). Untargeted formulations which largely depend on passive permeation through barriers do not meet the demand. Conversely, functional engineering of the drug delivery systems is the key future research trend for better delivery efficiency. Active targeting strategy can not only diminish the toxic or side effects due to the large dose, but also improve the drug concentration and extend the period of effective drug concentration at the targeted site, which is basically the organ of Corti. By exploring smart drug delivery systems with responsiveness to internal or external stimuli, researchers enable more precisely controlled drug release profile, or more robust drug entry propelled by those systems. We will next introduce those two major categories of advanced drug delivery strategy in NIHL therapies: targeted and responsive drug delivery, which are technically facilitated by biocompatible materials in their various forms.

#### Targeted drug delivery strategies

**Figure 8:**
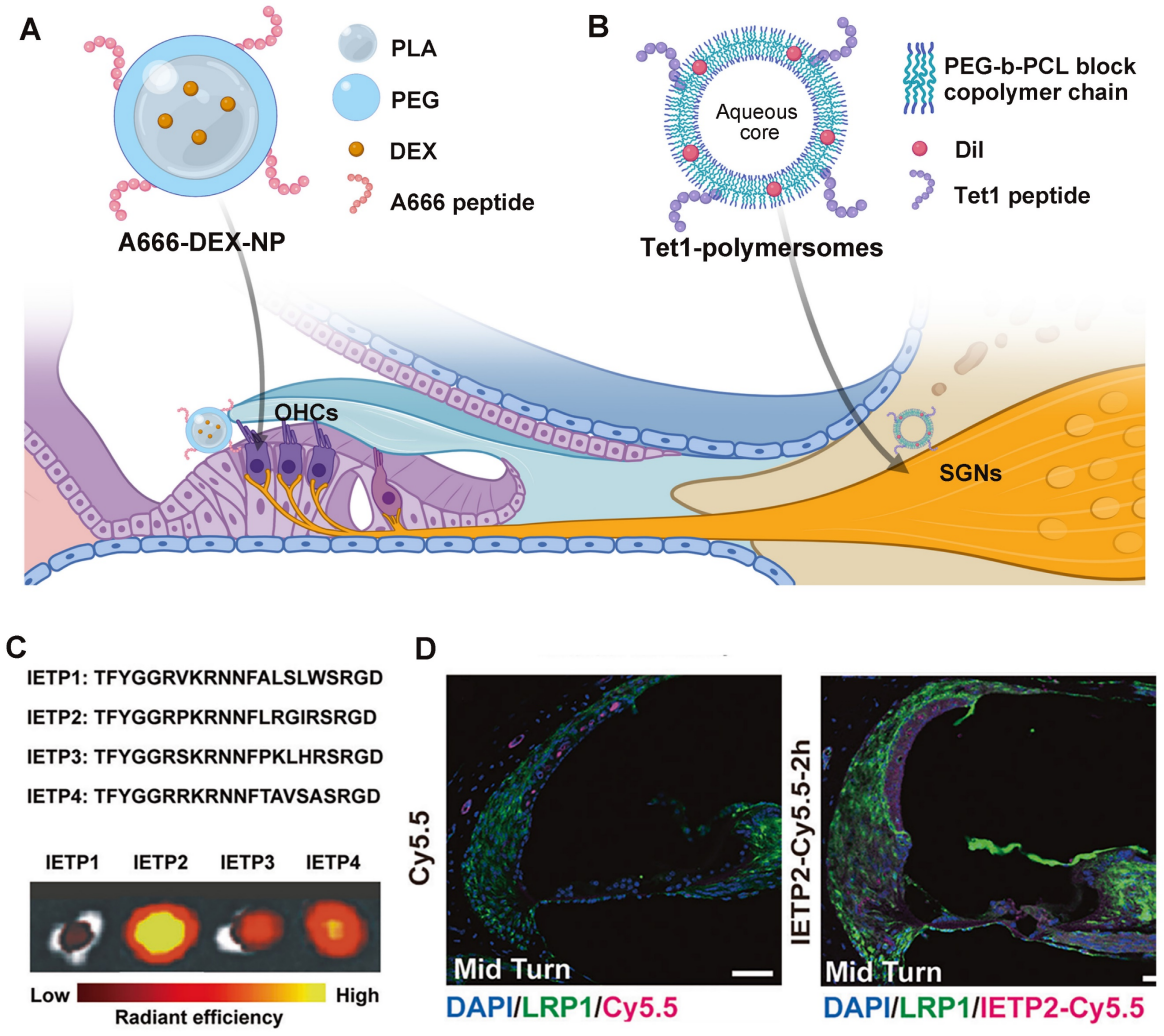
**Targeted drug delivery strategies for NIHL treatment. (A)** Schematic of the A666 peptide-conjugated nanodelivery system targeting prestin on OHCs 176. **(B)** Schematic of the Tet1 functionalized polymersome targeting the trisialoganglioside clostridial toxin receptors on SGNs 177. Created with BioRender (www.biorender.com). **(C)** Four candidate BLB targeting peptides and their uptake in mouse cochleae by *ex vivo* fluorescence imaging. Reproduced under the terms of the CC-BY Creative Commons Attribution 4.0 International License (http://creativecommons.org/licenses/by/4.0/) 127. Copyright 2022, The authors, published by Springer Nature. **(D)** The ability of IETP2 to cross the BLB *in vivo*. Reproduced under the terms of the CC-BY Creative Commons Attribution 4.0 International License (http://creativecommons.org/licenses/by/4.0/) 127. Copyright 2022, The Authors, published by Springer Nature. Abbreviations: OHCs: outer hair cells; SGNs: spiral ganglion cells; LRP1: low-density lipoprotein receptor-related protein 1

After a typical non-specific intratympanic drug delivery, there will be a decreasing basal-apical gradient of drugs or nanocarriers due to their slow diffusion within the fluid of the inner ear 105. It should be noted that with cochlear aqueduct connecting perilymph and cerebrospinal fluid 137, drugs or nanocarriers after intratympanic administration can be present in cerebrospinal fluid, posing potential off-target threats to the central nervous system 173. To minimize off-target effect and improve therapeutic outcomes, recognition units, such as ligands, aptamers and antibodies, were conjugated with drugs or nanocarriers to actively target certain cell types (Figure 8).

##### Targeting outer hair cells

Current targeted drug delivery for NIHL is focusing on prestin-binding peptides for OHC targeting. This is because prestin is the only recognized biomarker exclusively expressed in one single cochlear cell type 174, and hair cell damage is believed to play a critical role in the pathology of NIHL. After the identification of several prestin-binding peptides from phage display experiments 175, Kayyali et al. used one of the peptides to decorate liposomes, which improved the cochlear distribution of the nanocarriers to apical turns. With the payload of JNK inhibitor, the system produced a dramatic improvement in therapeutic outcomes than untargeted ones after an acute noise insult 105. Another two researches utilized the same peptide with appropriate modifications of the amino acid sequence in the construction of targeting nanocarriers against NIHL 164,165. The presence of peptide modification in functionalized nanocarriers significantly improved their protective effect against noise-induced hair cell apoptosis and hearing threshold elevation. Previously, our research group used another peptide designated, A666, to endow PEG6K-b-PCL19K polymersomes with OHC targeting ability (Figure 8A), which significantly improved the efficacy of DEX in a drug-induced ototoxicity model 176. So far, researchers have verified the selective accumulation of prestin-targeting nanoparticles in the cochlea of guinea pigs and C57BL/6J mice, and in the lateral line neuromasts of zebrafish. The major concern of this targeting system is that the expression level of prestin will drop sharply when the apoptosis of OHCs is set off, and therefore application within the therapeutic window is of critical importance.

##### Targeting spiral ganglion neurons

Besides hair cell loss, acoustic damage to neurons can also take part in the permanent hearing impairment. Specifically, synaptic trauma may lead to temporary loss of hearing, or more elusive perceptual disfunction 15. However, targeted drug delivery to the neural element of the cochlea has not been achieved in the management of NIHL, nor in that of other acquired cochlear disorders. Some neuronal protein biomarkers have shown significant potential among others, including the trisialoganglioside clostridial toxin receptor (GT1b) and the BDNF/NT-3 growth factors receptor (TrkB). Although their expression extends beyond the cochlear SGNs and throughout the central and peripheral nervous system 178,179, the systemic side effect can be minimized via local administration. A notable difference between the two candidates is that GT1b binding does not alter cell signaling, but TrkB activation contributes to a series of neuronal survival signaling. Targeting TrkB via peptide ligands or agonistic antibodies might at the same time enhance neurite survival 180. Whereas, the challenge of this system is that the specific binding with TrkB receptor does not necessarily lead to enhanced cellular uptake 181. The utility of GT1b and TrkB targeting strategies have been investigated in the development of inner ear drug delivery vehicles (Figure 8B) 177,181, showing translational potential in treating noise-induced neuropathy.

##### Targeting the blood-labyrinth barrier

For systemic drug delivery to the inner ear, targeting the BLB barrier is an emerging strategy. It does not aim to deliver therapeutic agents directly to the site of action, instead it improves the drug accumulation and transportation at the biological barrier. This is well described in delivering therapeutics to the CNS across the blood-brain barrier, but is still in its infancy in inner ear drug delivery 129. The utility of low-density lipoprotein receptor-related protein 1 (LRP-1) was initially explored in the drug delivery into the brain 182. Shi et al. first described the wide expression of LRP-1 on the BLB, as a receptor able to transport its multiple binding peptides across the BLB via endocytosis (Figure 8C and 8D) 127. By utilizing LRP-1 binding peptide, the study validated the efficient delivery of small-molecule compounds including potential drugs and imaging agents into the inner ear.

#### Responsive drug delivery strategies

Researchers have attempted to design or make use of various types of internal (e.g., pH, ROS, enzyme) and external (e.g., light, heat, magnetic field) stimuli to manipulate the *in vivo* behavior of drug delivery systems. These stimuli act either to promote RWM retention and penetration, or as a trigger for precise drug release at the site of action in the cochlea. Due to their versatility, responsive drug delivery systems have been intensively developed and employed mainly via an intratympanic delivery route in the research field of NIHL.

##### Thermal- and photosensitive gelling system

**Figure 9:**
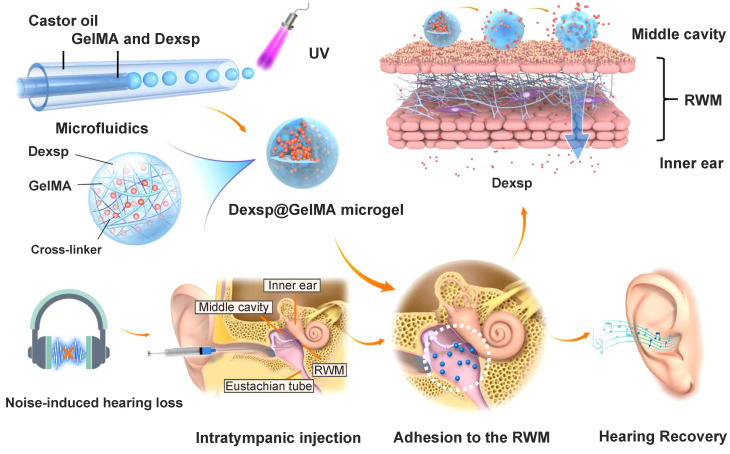
Schematics of the ultraviolet polymerized GelMA microgels for prolonged drug delivery of dexamethasone sodium phosphate for NIHL therapy. Reproduced with permission 143. Copyright 2022, American Chemical Society.

The stable and prolonged drug delivery on the RWM is always the essential purpose of using responsive gelling systems. They are more easily administered as a liquid, and then experience a sharp increase in their viscosity in response to the temperature change or ultraviolet (UV) light exposure. Poloxamer and chitosan are the most investigated thermosensitive biomaterials in this field 183. Poloxamer 407 is a triblock copolymer composed of both hydrophilic and hydrophobic segments, which endow it with temperature-dependent self-assembling ability 184. As reported by Li et al., poloxamer 188 with a higher hydrophilic content was supplemented to poloxamer 407 to obtain a finely tuned gelling temperature closest to that of the middle ear 170. Their utility has been well described in many NIHL preclinical researches, and innovative formulations such as gelatin methacryloyl (GelMA) hydrogel have great potential for future research. GelMA solution can be applied on the TM or RWM, and a UV light exposure triggers the covalent crosslinking process to form bulk GelMA hydrogels 185. Contrary to the bulk hydrogels, GelMA microgels do not impede the hearing with a micro-scale diameter, and they have been investigated recently as an otic drug delivery vehicle for NIHL therapy (Figure 9) 143. Researchers fabricated the microgels with an average diameter of 32.81 μm using a microfluidic method, and polymerized them by UV irradiation before adhesion to the RWM. Over 80% of the GelMA microgels remained closely attached to the RWM surface for one week, allowing the drug to be released in a sustained manner.

##### Ultrasound-aided microsystem

**Figure 10:**
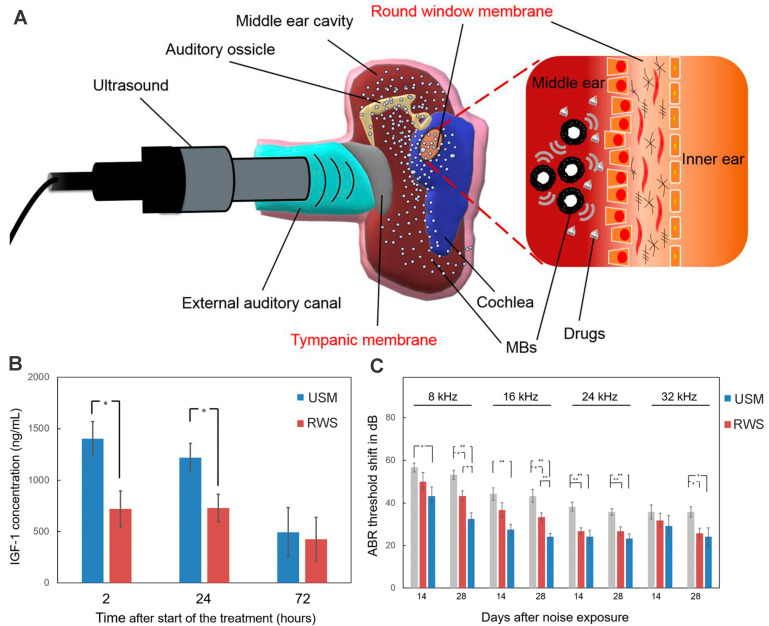
** (A)** Schematic of ultrasound-induced microbubble cavitation to increase the permeability of the RWM. Reproduced under the terms of the CC-BY Creative Commons Attribution 4.0 International License (http://creativecommons.org/licenses/by/4.0/) 187. Copyright 2020, The Authors, published by The American Society for Clinical Investigation. **(B)** Concentration of IGF-1 in the cochleae after the IGF-1 treatment with and without microbubble irradiation. Reproduced under the terms and conditions of the Creative Commons Attribution (CC BY) license (https://creativecommons.org/licenses/by/4.0/) 172. Copyright 2021, by The Authors, published by MDPI.** (C)** ABR threshold shift of guinea pigs after the IGF-1 treatment with and without microbubble irradiation. Reproduced under the terms and conditions of the Creative Commons Attribution (CC BY) license (https://creativecommons.org/licenses/by/4.0/) 172. Copyright 2021, by The Authors, published by MDPI. Abbreviations: MBs: microbubbles; RWS, round window soaking; USM, ultrasound microbubble treatment.

Ultrasound-driven microbubbles were applied to facilitate drug entry through the RWM (Figure 10A). Its theoretical basis is that the ultrasound-irradiated microbubbles can generate transient and reversible changes in the permeability of cell membrane via cavitation or sonoporation effect 186. After the RWM administration of a microbubble/DEX mixture followed by ultrasound irradiation, the DEX accumulation in perilymph was 2.4 to 11.2 times higher than that of round window soaking 71. Even if the drug was administered after the microbubble irradiation was finished, the inner ear delivery of rhIGF-1 was enhanced by 1.95 folds (Figure 10B). The two different therapies both resulted in significantly improved therapeutic effects for NIHL prevention and protection in guinea pigs 71,172.

##### Magnetically Driven nanoparticles

Magnetic particle-based approaches are naturally suitable for active and directional delivery of drugs from the tympanic cavity into the inner ear. After being deposited onto the RWM or injected into the middle ear, the magnetic particles can be pushed from the identical side or be pulled from the contralateral side into the inner ear with a powerful magnet 188,189. For example, superparamagnetic iron oxide nanoparticles (SPION) were attached to the AAV2 virus vector for the magnetic delivery of BDNF-gene therapy in a NIHL rat model. An additional advantage of SPION-based system is that their dynamic distribution in the cochlea can be visualized using MRI. Local application of this system substantially enhanced BDNF gene expression in the inner ear and reduced cochlear synaptopathy, with no ototoxicity to the inner ear 166.

Aside from the magnetic force, other kinds of physical fuels, or biofuels and chemical fuels, can be taken advantage of 190. For instance, Liang, et al. constructed a "slim waist" shaped microshotgun drug delivery system loaded with dry chemical propellants and nanoparticles for TM penetration 191. Here, water was the external stimuli added to ignite the chemical reaction inside the microshotgun, generating gas that ejected the nanoparticles deep into the TM. With Fe_3_O_4_ loaded in both the microshotgun wall and nanoparticles, a magnetic field can be applied to guide the microshotgun and provide the second penetration power directionally. Contrary to a usual tympanic injection, the system is a non-invasive intratympanic approach with minimum TM damage.

##### ROS-responsive nanoparticles

**Figure 11:**
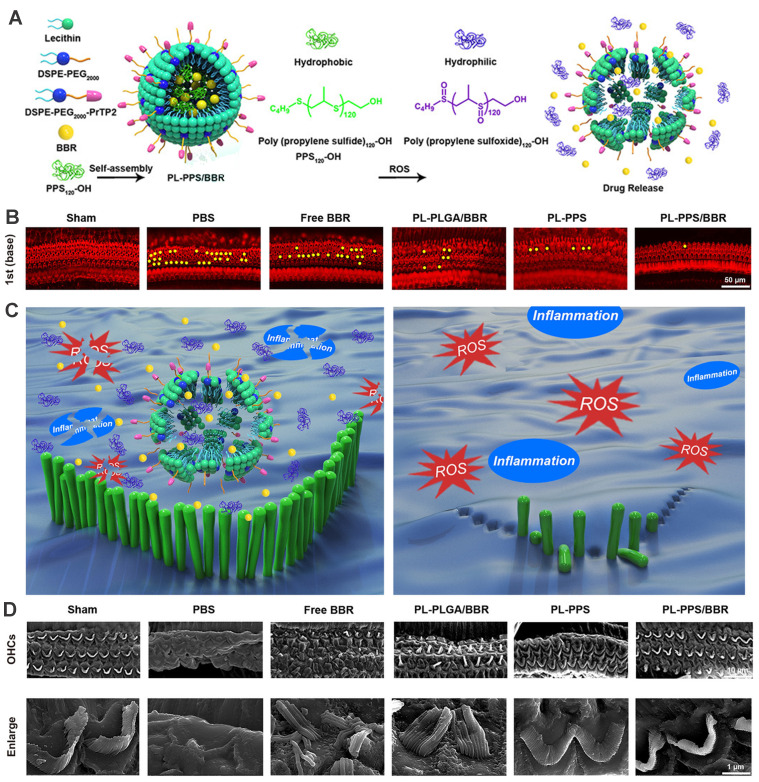
**(A)** Schematics of the ROS-Responsive nanoparticle (PL-PPS/BBR) delivery system which disassembled in a ROS-rich lymph for berberine release. **(B)** Comparison of OHC loss at basal turn in each group. **(C)** Schematics of the anti-inflammatory and antioxidant effects of PL-PPS/BBR on OHCs. **(D)** SEM images of OHCs and their stereocilia in each group. Reproduced with permission 164. Copyright 2021, American Chemical Society.

Considering that overproduction of ROS in various cochlear cell types was recognized as a crucial contributing factor to NIHL, the need for controllable and 'on demand' payload release at the ROS-rich sites was fueled. Poly(propylene sulfide) (PPS) is a ROS-receptor and a ROS-consuming material, which can be exploited to deliver drugs precisely and exert a scavenging effect on ROS 192. PPS can convert into more hydrophilic poly (propylene sulfoxide) and poly (propylene sulfone) and provoke the disintegration of the nanocarrier with rapid release of encapsulated cargo 193. Our group previously has developed a novel ROS-responsive/consuming nanoparticle system based on PPS, which combined the ROS scavenging effect of PPS with the antioxidant action of astaxanthin (ATX). The results of the study showed superior protective efficacy of the ATX-loaded PPS-NP than that of equivalent ATX in HEI-OC1 cell line, cochlear explants and guinea pigs 194. Zhao et al. integrated poly(propylene sulfide)120 (PPS120) into the OHC-targeting liposomes for ROS-responsive drug release in NIHL treatment (Figure 11) 164. *In vitro* H_2_O_2_-responsive drug releasing study suggested that PPS120 promoted berberine release once in the ROS environment, and exhibited greater ROS scavenging ability itself than berberine, leading to OHC morphological resilience and attenuated hearing loss to noise impairment *in vivo* 164.

## Challenges and future opportunities

Here, we provide a comprehensive and systematic overview of pathogenic mechanisms and therapeutic interventions regarding NIHL. Our understanding of the disease and means for intervention have been rapidly evolving, but several scientific issues related to NIHL design and clinical translation still require elucidation.

To begin with, traditional diagnostic methods are unmet for the expansion of NIHL, including noise-induced synaptopathy, noise-induced hidden hearing loss, and coding-in-noise deficit. The therapeutic interventions for noise-induced synaptopathy have been explored, but those evidences are primarily derived from morphological observations in animal models. There is no reliable method for evaluating the functional outcomes of these synaptic deficits in animals. Due to ethical limitations, it is nearly impossible to have direct histological observation in human subjects. The current diagnostic tools, which were mostly made to deal with NIHL caused by steady broadband noise, are inadequate to diagnose NIHL caused by different types of exposure. Therefore, it is urgent to provide guidelines for the methods that are recommended for assessment.

The development of novel drug targets and therapeutic molecules are in great need. Although progresses in understanding the mechanisms underlying NIHL have been made, little is known about the protective responses against noise and the cochlear repair after damage. It is speculated that the repair process may involve multiple cell types, including hair cells, supporting cells, fibrocytes in the lateral wall and migrated macrocytes. Further in-depth researches are needed to fully unleash the power of the innate defense and repair systems. Drug evaluations in animal models and later in clinical trials are challenging yet critical work because of the biological differences between *in vivo* and *in vitro* environments, and between different species.

It is well known that an appropriate drug delivery strategy can improve therapy outcomes and minimize side effects. The BLB or the RWM is the major challenge of drug delivery, depending on the route of administration chosen. We have discussed several potential strategies to open new frontiers in systemic delivery, but indeed there is a lack of clear, robust evidence to reveal the underlying mechanisms of the substance transport through the BLB, leaving very limited approaches available for opening the barrier. The intratympanic delivery, which has demonstrated superior efficacy in animal studies, relies on the use of advanced delivery systems including nanoparticles and gelling systems. Many of them have proven their utility as potential delivery platforms across barriers, but have not yet been applied in NIHL studies, such as the microshotgun drug delivery system. For more accurate delivery, it is essential to identify cell-specific protein biomarkers. Those biomarkers have to be expressed on the cell membrane and induce endocytosis upon ligand coupling. Only a few of them, including prestin, are considered as the most acknowledged candidates to meet such criteria. It is necessary to better our understanding of the major physicochemical parameters that mediate nanoparticle passage, biodistribution, and safety in cochlear fluids for drug delivery that based on biomaterials. In many NIHL researches, the assessment of the drug concentration and drug distribution once they enter the cochlea is omitted. Whether they have successfully overcome the tissue barrier is unclear and can only be speculated from their therapeutic effect. With prudence, the immunogenicity of biomaterials and the potential effects of advanced strategies such as targeting peptides should be evaluated.

**Figure 12:**
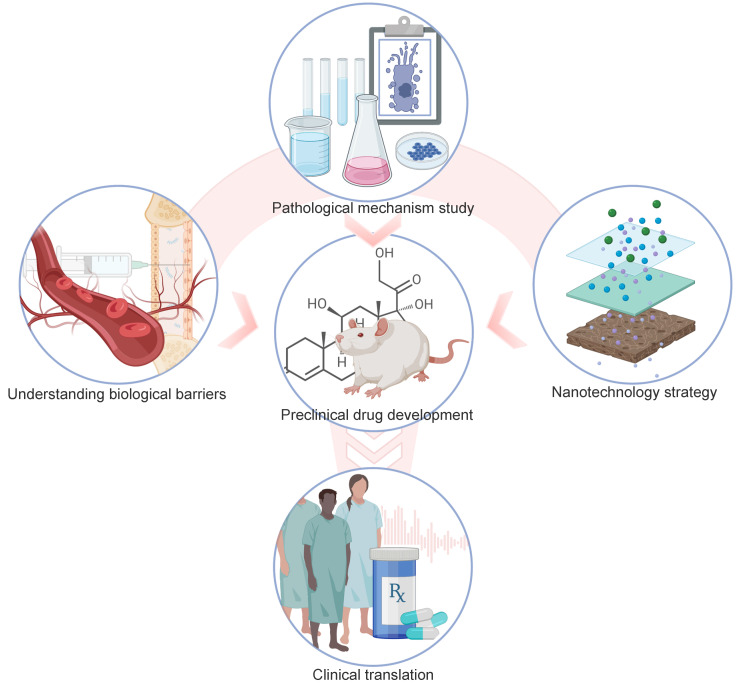
** Summary of drug development and drug delivery for NIHL.** Pathological mechanism studies of NIHL are the basics of preclinical drug development. More efficient drug delivery into the inner ear will be propelled by an advanced understanding of the main patterns of substance transport across the biological barriers, with the combined efforts of fast-developing nanotechnology. Those factors also contribute to the clinical translation of drug candidates. Created with BioRender (www.biorender.com).

Data acquired from animal models may have difficulty translating to human beings. There are huge differences in noise exposures experienced by preclinical animals and human subjects. The noise used in animal research, usually being wide or narrow band noise at a fixed sound pressure, is different from what people experience in real life, which is more complicated and flexible in pitch and intensity. It may have occurred in an intermittent pattern for years. As shown in Figure 12, future development of NIHL therapy relies on novel drug targets identified in NIHL mechanism studies, approaches to overcome biological barriers, and construction of advanced drug delivery systems. Distinguishing between different noise types and optimizing drug dose and time may better facilitate the translation of basic NIHL research to the clinical setting.

## Figures and Tables

**Table 1:** Drug delivery systems in NIHL animal models

Drug delivery systems	Characteristics	Drug	Pharmacological action	Animals	Noise	Administration	Therapeutic outcomes	Ref.
PEGylated PLA nanoparticles	116 nm	Betamethasone phosphate	Anti-inflammatory	CBA/N mice4-6 weeks	OBN 120 dB SPL 8 kHz 2 h	IVImmediately after noise	GR translocation into the nuclei in OHCs↓ABR TS at 8 kHz on day 14, 32 kHz on days 7 and 14↓OHC loss in the apical turns; ↓IHC loss in the apical and basal turns	131
Solid lipid nanoparticles	Not described	Edaravone	Antioxidant	Guinea pigsAdult	SN 110 dB SPL 0.25-4 kHz 2 h/d, 4 d	ITI1 d after noise	↓ABR TS on day 4↓ROS in cochlea	161
Zeolitic imidazolate framework-based nanoparticles	105 nm PDI = 0.22 Zeta potential = -6.9 mV	Methylprednisolone	Anti-inflammatory	C57BL/6J mice4-5 weeks	WN 100 dB SPL 3 h/d, 3 d	IVBefore noise	↓ABR TS at 16 kHz, 24 kHz, and 32 kHz↓HC loss at basal and middle turns	130
Solid lipid nanoparticles	203 nmPDI=0.361	Clozapine	Antioxidant	Guinea pigsAdult	SN 110 dB SPL 0.25 - 4 kHz 2 h/d, 4 d	ITIImmediately after noise	↓ABR TS on day 6↓ROS in cochlea	162
Cross-linked hyaluronic acid with polylactide-*co*-glycolide microcapsules	50 μm	Dexamethasone;Insulin-like growth factor 1	Anti-inflammatory;Anti-apoptosis; Inducing the proliferation of supporting cells	SD Rats6 weeks	WN 3.7 h	ITI 3 h after noise	↓ABR TS	163
ROS-responsive and OHC-targeting liposomes	219.08 ± 8.86 nmZeta potential = -6.70 ± 0.13 mV	Poly(propylenesulfide)120;Berberine	Anti-inflammatory and antioxidant	Guinea pigs10-12 weeks	WN 120 dB SPL 6.3-20 kHz 4 h	RWM application 12 h after noise	↓MDA and ↑SOD ↓TNF-α, IL-6 and IL-1↓OHC loss in four turns and better morphology of OHCs↓ABR TS at 8, 16 and 24 kHz on days 4, 7 and 14	164
OHC-targeting liposomes	116.6 nmzeta potential = - 18.58 mV	forskolin	Antioxidant	C57BL/6J mice	WN 115 dB SPL 2-20 kHz 2 h	RWM application3 d before noise	↓ABR TS at 4, 8 and 16 kHz on days 1, 7 and 14; 32 kHz on day 28↓OHC loss in the mid-basal turns	165
Superparamagnetic iron oxide nanoparticles	magnetically driven	AAV2-BDNF vectors	Promoting the SGNs survival	Long-Evans rats6 - 8 weeks	OBN110 dB SPL8-16 kHz 2 h	RWM applicationMagnet placed on the contralateral ear for 30 min3 d after noise	↓ABR TS at 16, 24 and 32 kHz by 2 weeks↓Decrease of ABR wave I amplitude↓Synaptic ribbon loss in the basal and mid turns ↑BDNF expression	166
Gelatin methacryloyl microgel particles	32.81 μmCoefficient of variation = 15%	Dexamethasone sodium phosphate	Anti-inflammatory	Guinea pigsAdult	WN 120 dB SPL 6 h	ITI1 d before noise	↓ABR TS at 4, 16, 24, 32 kHz↓OHC loss↓Synaptic ribbon loss↓Decrease of ABR wave I amplitude↓Increase of ABR wave I latency	143
Gelatin hydrogel	Biodegradable	Insulin-like growth factor 1	Anti-apoptosis; Inducing the proliferation of supporting cells	Guinea pigsAdult	OBN 120 dB SPL 4 kHz 5 h	RWM application 5 h after noise	↓ABR TS at 4 kHz on days 14 and 21; 8 kHz on day 14↓OHC loss in basal and middle turns	167
Gelatin hydrogel	Biodegradable	Hepatocyte growth factor	Anti-apoptosis	Guinea pigs4 weeks	OBN 120 dB SPL 4 kHz 3 h	RWM application 1 h after noise	↓ABR TS at 16 kHz on day 21↓OHC loss in the 60-80% distance from the apex	168
Chitosan glycerophosphate hydrogel containing PEGylated OHC-targeting liposomes	Liposomes: OHCs targeting 87 ± 5 nmGel: Chitosan glycerophosphate-based Thermosensitive	D-JNKi-1	Anti-apoptosis	CBA/J mice6-10 weeks	WN 115-120 dB SPL 6.3-20 kHz 4 h	RWM application 2 d before noise	↓ABR TS in 4, 8, 16, 24, 32 kHz in 1-14 days	105
Hyaluronic acid gel containing liposomes	Liposomes: 140 nmGel: Hyaluronic acid-based	Dexamethasone	Anti-inflammatory	Guinea pigs	PT 100 dB SPL 5 kHz 1 h	RWM application 2 d after noise	No additional hearing recovery at 4, 16, 24 and 32 kHz compared to spontaneous recovery	169
Thermosensative hydrogel containing sodium phosphate multivesicular liposomes	Liposomes: 14.7 μmGel: Poloxamer-based; Gelling temperature at 34.9 °C	Dexamethasone	Anti-inflammatory	Guinea pigs Adult	WN 112 dB SPL 20-20000 Hz 6 h	ITI1 d before noise	↓ABR TS at 4, 8, 16 and 24 kHz on day 7↓OHC loss in the basal and middle turns	170
Monodisperse droplets of water	6.96 μmFlow rate = 20 μL/min	Methylprednisolone	Anti-inflammatory	Guinea pigsAdult	WN 106 dB SPL 15-125 kHz 48 h	ITIImmediately after noise	↓ABR TS after 2 weeks↓OHC loss in 5 and 8 kHz frequency range	171
Ultrasound-aided microbubbles	7.09 μm10^8 bubbles/mL	Dexamethasone	Anti-inflammatory	Guinea pigs	WN 118 dB SLP 5 h	RWM application followed by ultrasound2 h before noise	↓ABR TS at 8, 16, and 32 kHz on days 7, 14, and 28↓OHC loss in the basal and second turns↓HMGB1 and ICAM-1 expression in spiral ligament	71
Ultrasound-aided microbubbles	0.7-10 μm 1*-*5 *×* 10^5^ bubbles/mL	Insulin-like growth factor 1	Anti-apoptosis; Inducing the proliferation of supporting cells	Guinea pigs	NBN 118 dB SPL 8 kHz 5 h	RWM application or ITI followed by ultrasound 24 h after noise	↓ABR TS at 8, 16 and 24 kHz on days 14 and 28; 32 kHz on day 28↓OHC loss in the basal and second turns ↓Synaptic ribbon loss in the basal and second turns ↑Akt1 and Mapk3 expression	172

Abbreviations: ABR: auditory brainstem response; AN: after noise; BN: before noise; GR: glucocorticoid receptor; HC: hair cell; HGF: hepatocyte growth factor; HMGB1: high mobility group box 1; ICAM-1: intercellular adhesion molecule-; IHC: inner hair cell; IL-1: interleukin 1; IL-6: interleukin 6; IP: intraperitoneal; IT: intratympanic; IV: intravenous; MDA: malondialdehyde; NBN: narrowband noise; NP: nanoparticle; OBN: octave band noise; OHC: outer hair cell; PDI: polydispersity index; PEG: polyethylene glycol-coated; PLA: polylactic acid; PT: pure tone; rhIGF-1: recombinant human insulin-like growth factor 1; ROS: reactive oxygen species; RWM: round window membrane; SN: stationary noise; SPL: sound pressure level; SOD: superoxide dismutase; TNF-α: tumor necrosis factor; TS: threshold shift; WN: white noise

## Abbreviations

AMPK: adenosine monophosphate-activated protein kinase
BLB: blood-labyrinth barrier
CPEs: chemical permeation enhancers
eNOS: endothelial nitric oxide synthases
GelMA: gelatin methacryloyl
GT1b: trisialoganglioside clostridial toxin receptor
Glu: glutamate
GluRs: glutamate receptors
HMGB1: high mobility group box 1
IHCs: inner hair cells
IL-1β: interleukin-1beta
IL-6: interleukin-6
JNK: c-Jun N-terminal kinase
LRP-1: low-density lipoprotein receptor-related protein 1
MAPK: mitogen-activated protein kinase
NAC: N-acetylcysteine
NIHL: noise-induced hearing loss
NO: nitric oxide
NPs: nanoparticles
NRF2: nuclear factor erythroid2-related factor 2
OHCs: outer hair cells
PKM2: pyruvate kinase M2
PPS: poly(propylene sulfide)
RWM: round window membrane
ROS: reactive oxygen species
SGNs: type I spiral ganglion neurons
SIRT: sirtuin
SPION: superparamagnetic iron oxide nanoparticles
TDNs: tetrahedral DNA nanostructures
TM: tympanic membrane
TNF-α: tumor necrosis factor-alpha
VGCCs: voltage-gated Ca^2+^ channels
